# Molecular Detection of Multiple Emerging Pathogens in Sputa from Cystic Fibrosis Patients

**DOI:** 10.1371/journal.pone.0002908

**Published:** 2008-08-06

**Authors:** Fadi Bittar, Hervé Richet, Jean-Christophe Dubus, Martine Reynaud-Gaubert, Nathalie Stremler, Jacques Sarles, Didier Raoult, Jean-Marc Rolain

**Affiliations:** 1 Unité de Recherche sur les Maladies Infectieuses et Tropicales Emergentes (URMITE), CNRS-IRD, UMR 6236, Faculté de Médecine et de Pharmacie, Université de la Méditerranée, Marseille, France; 2 Département des Maladies Respiratoires, Centre de Ressources et de Compétences pour la Mucoviscidose Enfants (CRCM), Hôpital Timone, Marseille, France; 3 Département des Maladies Respiratoires, Centre de Ressources et de Compétences pour la Mucoviscidose Adultes (CRCM), Hôpital Sainte Marguerite, Marseille, France; The Research Institute for Children at Children's Hospital New Orleans, United States of America

## Abstract

**Background:**

There is strong evidence that culture-based methods detect only a small proportion of bacteria present in the respiratory tracts of cystic fibrosis (CF) patients.

**Methodology/Principal Findings:**

Standard microbiological culture and phenotypic identification of bacteria in sputa from CF patients have been compared to molecular methods by the use of 16S rDNA amplification, cloning and sequencing. Twenty-five sputa from CF patients were cultured that yield 33 isolates (13 species) known to be pathogens during CF. For molecular cloning, 760 clones were sequenced (7.2±3.9 species/sputum), and 53 different bacterial species were identified including 16 species of anaerobes (30%). Discrepancies between culture and molecular data were numerous and demonstrate that accurate identification remains challenging. New or emerging bacteria not or rarely reported in CF patients were detected including *Dolosigranulum pigrum*, *Dialister pneumosintes*, and *Inquilinus limosus*.

**Conclusions/Significance:**

Our results demonstrate the complex microbial community in sputa from CF patients, especially anaerobic bacteria that are probably an underestimated cause of CF lung pathology. Metagenomic analysis is urgently needed to better understand those complex communities in CF pulmonary infections.

## Introduction

Cystic fibrosis (CF) is an autosomal recessive disorder due to mutations in the Cystic Fibrosis Transmembrane conductance Regulator (CFTR) gene that is located on chromosome 7 in human [1]. The bronchopulmonary infections represent the major problem in CF patients that lead to a decrease of the lung function and are responsible of a high morbidity and mortality [2]. Although the life expectancy of these patients has increased over last years, the mortality remains high in patients between 26 and 30 years. It has been demonstrated that accurate antimicrobial treatment are of great importance to avoid a rapid destruction of the lung functions and the spread of multidrug-resistant and/or highly virulent pathogens [3]. Nevertheless, accurate isolation and identification of bacteria from sputa of CF patients is often difficult. Indeed, conventional methods used for isolation of bacteria from sputa usually consist of the use of selective media [4] adapted to the culture of several pathogens mainly *Staphylococcus aureus*, *Pseudomonas aeruginosa*, *Streptococcus pneumoniae*, *Haemophilus influenzae*, and *Burkholderia cepacia*. However, these media are not able to isolate fastidious or emerging bacteria and are often contaminated with mixed bacteria and/or fungi. Moreover, mucoid *P. aeruginosa* usually invades all the agar plates so that it becomes impossible to isolate other bacteria [5]. It also has been documented that *P. aeruginosa* can produce substances that inhibit the growth of other bacteria [5]. Finally, even if bacteria are isolated, correct identification is often difficult due to either the phenotypic variations as frequently reported for *P. aeruginosa* (lack of pigments) [6], *S. aureus* (small colony variants) [7], and *B. cepacia* [8] or the fact that some bacteria are not recorded in the databases of the commercial phenotypic systems currently available.

Molecular biology techniques for correct detection and identification of bacteria is now widely used in clinical microbiology and also developed for identification of isolates obtained from CF patients especially for gram-negative nonfermenting bacilli by the use of 16S rDNA gene sequencing [6], [8]. Although these techniques can identify precisely isolated bacteria, they can not evaluate bacterial diversity in polymicrobial samples. Other approaches have been used to identify these bacterial communities, mainly based on analysis of the sputa by terminal restriction fragment length polymorphism (RFLP) using the 16S rRNA target gene [4] or using specific hybridization probes methods [5]. Nevertheless, these techniques present limits, as it can not identify new or unknown pathogens [4], [5].

The primary purpose of this study was to compare the phenotypic identification of bacteria to molecular methods using 16S rDNA amplification, cloning, and sequencing in a series of sputa from CF patients. The secondary purpose was to evaluate the bacterial diversity and to describe new or emerging pathogens colonizing CF lung.

## Results

### Samples

During a 2 months period (December 2005–January 2006) we have collected 25 sputa from 16 children (8.9 years±5.9) and 9 adults (29 years±9.4).

### Bacterial culture and identification of the isolates

**Figure 1:**
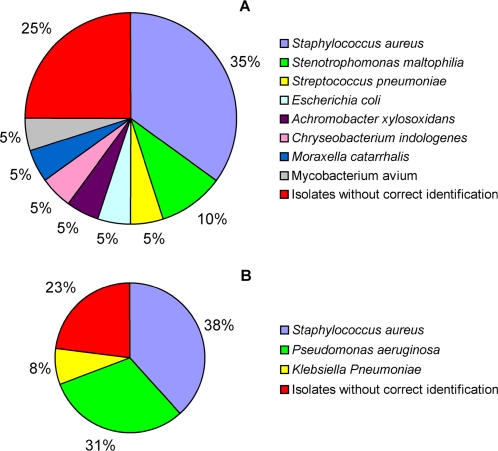
Bacteria identified by the conventional culture methods in children (A) and in adults (B).

Among the 16 sputa from children, 20 known pathogens were isolated from 14 sputa whereas mixed oropharyngeal flora was retrieved from 2 patients (patient 3 and 7). The conventional phenotypic identification was as follows: 7 *S. aureus*, 2 *Stenotrophomonas maltophilia*, 1 *S. pneumoniae*, 1 *Escherichia coli*, 1 *Achromobacter xylosoxidans*, 1 *Chryseobacterium indologenes*, 1 *Moraxella catarrhalis*, 1 *Mycobacterium avium* (a positive isolate with Ziehl-Neelsen coloration), and 5 (n = 5/20, 25%) isolates without correct identification (Fig. 1A). Among the 9 sputa from adults, 13 bacteria were isolated including 5 *S. aureus*, 4 *P. aeruginosa*, 1 *Klebsiella pneumoniae*, and 3 (23%) isolates without correct identification (Fig. 1B). Correct identification for the 8 atypical isolates was achieved after partial sequencing of 16S rRNA gene leading to *S. pneumoniae*, 2 *Inquilinus limosus*, *A. xylosoxidans*, *Serratia marcescens*, *S. aureus*, *Burkholderia multivorans*, and *P. aeruginosa* (Table 1).

**Table 1:** Genotypic identification of the 8 misidentified isolates.

Patient No.	Age (year)	Phenotypic identification (%) API system	16S rRNA sequencing (GenBank accession number, %)
4	1	*Streptococcus salivarius* (99.8%)	*Streptococcus pneumoniae* (AE008546, 96%)
5	2	*Sphingomonas paucimobilis* (99.8%)*	*Inquilinus limosus* (AY043375, 99%)
9	12	*Achromobacter xylosoxidans* (49.5%)*	*Achromobacter xylosoxidans* (AF411020, 100%)
11	13	*Burkholderia cepacia* (87.7%)*	*Serratia marcescens* (AJ550467, 99%)
16	17	*Agrobacterium radiobacter* (97.7%)*	*Inquilinus limosus* (AY043375, 99%)
17	18	Orange gram-positive coccus*	*Staphylococcus aureus* (BX571857, 99%)
19	19	*Pseudomonas stutzeri* (95%)*	*Burkholderia multivorans* (Y18703, 99%)
22	30	Multiresistant gram-negative coccobacilli grew on chocolate Poly ViteX agar but not on MacConKey agar	*Pseudomonas aeruginosa* (AY631241, 99%)

* isolate grew on Cepacia agar.

### Analysis of sputa after PCR and cloning

A total of 1,000 clones containing the correct size of the insert (approximately 1,230 bp) were collected from the 25 sputa. Finally we have sequenced 760 clones (24 clones for 15 patients and 40 clones for 10 patients) and 736 exploitable bacterial sequences (96.8%) were analyzed. All obtained sequences have a sequence similarity score of ≥97% when compared with the existing sequences in the GenBank database except 11 sequences including 4 *S. pneumoniae* (AE008546, 96%), 3 *Streptococcus constellatus* (AY277939, 94%), 2 *Streptococcus iniae* (AF335573, 95%), 1 *H. influenzae* (AY613741, 91%) and 1 *A. xylosoxidans* (AF531768, 95%). These 11 sequences may be considered as being obtained from putative new species.

**Figure 2:**
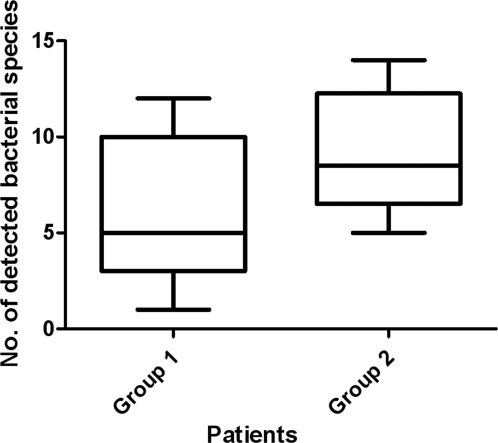
Box plot graph representing the number of detected bacterial species in the two groups of patients. Group 1 = patients to whom 24 clones from their sputa have been sequenced; Group 2 = patients to whom 40 clones from their sputa have been sequenced.

The 736 sequences corresponded to 53 different bacterial species that are listed in Table 2. The mean number of bacterial species per sputum was 7.2 bacteria ±3.9 [range 1–14] (7.2±3.5 for the children and 7.2±4.7 for the adults). The mean number of detected species was statistically higher when the number of sequenced clones was increased (6.0±4.0 species for 24 sequenced clones versus 9.0±3.1 species for 40 sequenced clones) (p = 0.05) (Figure 2). Of these 53 species, 9 were previously reported as pathogen in CF patients and represented 344 clones (46.7%) (Table 2). The other species were either bacteria from oral flora, especially anaerobic bacteria, or bacteria detected in endodontic/peridontic infections, or bacteria of unknown pathogenicity (392 clones, 53.3%). The anaerobes represented 30.2% of the detected species (16/53 species) and 16.2% of the total sequenced clones (119/736 clones) (Table 2).

**Table 2:** The bacterial diversity detected using the genomic methods.

Pathogen species	No. of clones	Other species	No. of clones
*Achromobacter xylosoxidans* *	9	*Abiotrophia defectiva* *	1
*Burkholderia multivorans* *	11	*Actinomyces* sp.*	2
*Haemophilus influenzae* *	7	*Bergeyella* sp.†	1
*Moraxella catarrhalis* †	20	*Capnocytophaga* sp.*	1
*Pseudomonas aeruginosa* *	99	*Carnobacterium* sp.†	4
*Serratia marcescens* †	3	*Dolosigranulum pigrum* †	8
*Staphylococcus aureus* *	129	*Eikenella corrodens* * †	2
*Stenotrophomonas maltophilia* *	41	*Escherichia coli* *	2
*Streptococcus pneumoniae* *	25	*Gemella haemolysans* * †	20
**Total (%)**	344 (46.7%)	*Gemella sanguinis* *	2
		*Granulicatella adiacens* *	5
**Anaerobic species**	**No. of clones**	*Granulicatella paradiacens* *	17
*Dialister pneumosintes* * †	1	*Kingella denitrificans* * †	1
*Gemella morbillorum* * †	4	*Kingella oralis* * †	2
*Lachnospiraceae genomosp.* * †	2	*Lactobacillus delbruekii* * †	1
*Peptostreptococcus* sp.*	1	*Neisseria* sp.*	33
*Porphyromonas* sp.*	20	*Rothia mucilaginosa* *	4
*Prevotella denticola* *	6	*Streptococcus anginosus* *	5
*Prevotella melaninogenica* *	12	*Streptococcus constellatus* * †	3
*Prevotella oris* *	2	*Streptococcus cristatus* *	1
*Prevotella salivae* *	1	*Streptococcus genomosp.* *	24
*Prevotella* sp.*	37	*Streptococcus gordonii* *	4
*Selenomonas infelix* * †	2	*Streptococcus iniae* †	2
*Selenomonas noxia* * †	1	*Streptococcus mitis* *	13
*Selenomonas* sp.*	4	*Streptococcus parasanguis* *	10
*Tannerella forsythensis* * †	2	*Streptococcus salivarius* *	20
*Veillonella atypica* *	1	*Streptococcus sanguinis* *	2
*Veillonella* sp.*	23	*Streptococcus* sp.*	83
**Total (%)**	119 (16.2%)	**Total (%)**	273 (37.1%)

* Species that have been detected either in normal oral flora, endodontic/peridontic infections, VAP, or CF samples using T-RFLP or cloning [4], [9], [11], [13]–[20].

† First species detection in CF samples using cloning in this study.

### Comparison between culture and cloning

**Figure 3:**
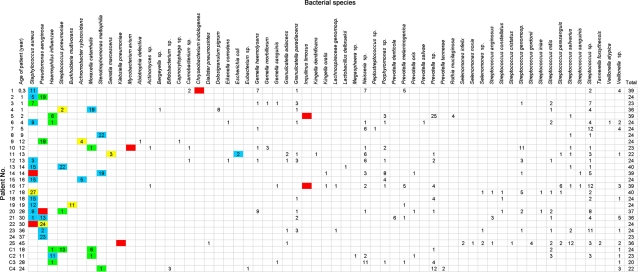
Comparison between phenotypic and genotypic detection and identification. The number in the box indicates the number of clones obtained for each bacterial species in each sputum. C1, C2, C3, and C4 were control analysis. Blue color box = concordant results between PCR-cloning and culture; red color box = negative PCR-cloning and positive culture; green color box = positive PCR-cloning and negative culture; yellow color box = misidentified bacteria.

Among the 33 isolated bacteria from culture, 19 bacteria (57.6%) were also found after PCR and cloning (blue boxes, Figure 3). Eight isolates (24.2%) were not found after PCR and cloning (red boxes, Figure 3) including: 2 *S. aureus*, 1 *P. aeruginosa*, 1 *K. pneumoniae*, 1 *M. avium*, 1 *C. indologenes* et 2 *I. limosus*. Interestingly, all these bacteria were recovered in very small quantity in culture (10^2^_−_ 10^3^ CFU/ml) and the cloning enabled us to detect many bacteria of the oral flora as well as anaerobes. Six isolates that were previously misidentified in culture from patients 4, 9, 11, 17, 19, and 22 could be detected after PCR and cloning and their identifications were 100% identical to that of those obtained from direct 16S rRNA sequence of isolates (Table 1 and yellow boxes, Figure 3).

Conversely, five pathogenic and theoretically cultivable bacterial species were only detected after PCR and cloning in 7 patients (green boxes, Figure 3) including 1 *S. aureus*, 2 *P. aeruginosa*, 2 *H. influenzae*, 1 *S. pneumoniae*, and 1 *M. catarrhalis*. Interestingly, these 7 patients were known to be chronically infected with these bacteria and all of them received adapted antimicrobial therapy prior to the analysis of their sputum sample. Conversely, for the other patients, they did not receive antibiotics prior to isolation of the bacteria in their sputa.

### Control analysis

In control group consisting of 4 patients with bronchiectasis (1 child and 3 adults), the culture was interpreted as mixed oropharyngeal flora for 3 patients and *H. influenzae* was isolated for one patient. PCR and cloning of these sputa and sequencing of 89 clones showed several pathogens such as *S. pneumoniae*, *H. influenzae*, *M. catarrhalis*, and *Stenotrophomonas maltophilia* besides the oral flora and anaerobes (Figure 3). The mean number of bacterial species per sputum in bronchiectasis group was 6.8 bacteria ±1 [range 6–8] versus 7.2 bacteria ±3.9 [range 1–14] in CF group (p = 0.8). Interestingly, the number of different species detected in CF group (53 species) was statistically higher than in control group (13 species) (p = 0.0004, Relative Risk = 1.8).

## Discussion

The conventional phenotypic techniques of isolation, identification, and evaluation of the antibiotic susceptibility for the pathogens in sputa from CF patients remain to date the base line in therapeutic management during the bronchial exacerbations of this disease. Nevertheless, the applied culture methods have been adapted to isolate the common bacteria known to be involved in bronchial infections of CF patients. So they neither allow to evaluate the microbial diversity of sputa, nor the detection of new bacteria which can participate in the physiopathology of the disease.

The bacterial diversity in a complex flora such as sputa from CF patients was previously studied using RFLP techniques [4], [9] or temporal temperature gradient gel electrophoresis (TTGE) followed by pyrosequencing [10] that are known to be time-consuming and are not able to identify new pathogens. Only three reports have described the amplification of 16S rRNA gene followed by cloning and sequencing to identify these complex flora but this was either for a very limited number of patients and a limited number of clones [4], [9] or only from children with CF [11]. This approach has been successfully used for identification of bacterial species for the diagnosis of other infections such as bone and joint infections [12], endodontic infections [13]–[15], periodontitis [16]–[18], and ventilator-associated pneumonia (VAP) [19] allowing the detection of fastidious and/or emerging pathogens. In our study, 57.6% of isolated bacteria were found only after cloning and sequencing. For the pathogenic species only detected after amplification and cloning (7 cases), patients had received an effective antibiotic therapy that can explain the negativity of the culture. It is important to know that the detection of bacteria by PCR does not necessarily reflect the viability of these bacteria. It is also possible that some bacteria can enter viable but uncultivable state. For the false-negative PCR cloning results, the load of bacteria on agar was low as compared to oropharyngeal flora, suggesting that the prevalent flora was amplified preferentially. Another explanation of these discordances could be a low specificity of the universal primers for some bacteria, especially for *I. limosus* and *M. avium*.

Finally, the analysis of sputa after PCR and cloning enabled us to obtain, besides the pathogens recovered from culture, 392 additional sequences identifying 44 different bacterial species including 5 putative new species. These species have been documented mainly in normal bacterial flora of the oral cavity [20] and often in endodontic/periodontic infections [13]–[18] or in VAP [19] (Table 2). Interestingly, clonal analysis of tongue and lung samples taken from patients with VAP revealed a wide range of bacterial diversity in the oral cavity and lung and indicated that the dorsal surface of the tongue serves as a potential reservoir for bacterial species involved in VAP [19]. In the case of CF, current knowledge shows that the pulmonary infections and bronchial exacerbations must be considered as polymicrobial infections [4], [5], [9], [10]. Recent works have shown that the bacterial diversity in sputa of CF patients was much more important than that previously considered [4], [5]. Rogers et al have recently compared bacterial communities in sputum and mouthwash samples from patients with CF using T-RFLP and have demonstrated that sputum from the lungs of these patients is not contaminated by bacteria present in the oral cavity and they have also provided evidence that the CF lung can be colonised by certain oral bacterial species [21]. The Table 2 presents the comparison of the species detected in our study in CF patients with those previously detected either in normal bacterial flora of the oral cavity [20], in endodontic/periodontic infections [13]–[18], in VAP [19], or in CF samples [4], [9], [11] using T-RFLP or 16S rDNA clone libraries analysis. Interestingly, we have found in these patients several pathogens not isolated in the culture besides the oral flora and anaerobes like *Prevotella* and *Porphyromonas* species.

In our study, we were able to detect 16 anaerobic bacterial species representing 119 of 736 clones (16.2%). It is not usual to look for or to isolate anaerobes from sputa of CF patients. A recent study has described the isolation of a range of anaerobic species in large numbers and from 64% of sputum samples taken from patients with CF [22] with the genera *Prevotella* and *Veillonella* being the most frequently isolated [22]. These two genera are also detected frequently in our work. Because anaerobic bacteria are naturally resistant to certain antibiotics including aminoglycosides [23] that are frequently used during CF, one can hypothesize that these anaerobes may be selected after an elective course of antibiotics therapy. In the study of Jewes et al., anaerobes were isolated in about 24% of tested samples during either bronchial exacerbations or not [24]. The proportion of the detected anaerobes in two other studies was 16.5% (17/103 clones) [4] and 26.4% (14/53 clones) [9]. The percentage of anaerobic bacteria in a recent study in CF patients ranged from 27 to 93% of the clones examined. Interestingly, such anaerobic bacteria were rare in CF subjects with typical pathogens [11]. Conversely, in another work, the colonisation with *P. aeruginsa* significantly increased the likelihood that anaerobic bacteria would be present in the CF sputum [22]. The role of these anaerobes in the physiopathology of CF remains unclear. However, recent data demonstrate that the oropharyngeal flora, that are not normally thought of as serious problems in CF, can induce a positive regulation (phenomenon of quorum sensing) of genes of virulence of *P. aeruginosa* [25]. Conversely, it is also possible that *P. aeruginosa* can influence virulence factor genes of secondary pathogens or those potentially present in the oropharyngeal flora strains [25] that gain in pathogenicity. Therefore oropharyngeal bacteria likely participate in disease progression and are probably an underestimated emerging cause of CF lung pathology that should be considered in the antibiotic strategy. This phenomenon of inter-cellular communications is also important in the constitution of the biofilms [26]. Moreover, many studies illustrate that the activity of several antibiotics including aminoglycosides and quinolones against *P. aeruginosa*, residing in a biofilm mode within hypoxic inspissated airway mucus and undergoing anaerobic metabolism, is significantly reduced or ineffective under anaerobic conditions than under aerobic conditions [27]–[29]. New therapeutic approaches by inhibition of the phenomenon of quorum sensing and anaerobes may be useful in the treatment of the respiratory infections in CF patients as recently demonstrated with azithromycin [30].

Finally, many bacteria identified in our work, like *I. limosus*, *G. adjacens*, *R. mucilaginosa*, *D. pneumosintes*, *D. pigrum* are not classically described in the expectoration of CF patients. In the study of Harris et al., potentially novel pathogens in CF subjects were also detected using rRNA gene libraries including the genus *Lysobacter*, members of the *Coxiellaceae* and *Rickettsiales*, *Prevotella denticola*, and *Streptococcus intermedius* [11]. Recently, *I. limosus* has been documented mainly in CF patients and was sometimes accompanied with exacerbation or respiratory decline [31]–[37]. This bacterium was described in 1999 [34] and was characterized in 2002 [31]. We think that the prevalence of this multi-resistant bacterium may be underestimated because of its recent description, its slow growth on selective media, its absence in the databases of commercial identification kits, and the need of molecular methods for its identification. *D. pigrum* was also recently reported in human respiratory diseases: a case of pneumonia [38], a case of VAP [39], and finally a case of nosocomial pneumonia and septicaemia [40]. Human infection diseases caused by *D. pneumosintes* (formerly *Bacteroides pneumosintes*) including supradiaphragmatic infections in the respiratory tract and brain abscesses were reviewed in recent study evaluated the antimicrobial susceptibility of large collection of *Dialister* clinical isolates [41]. Although the role of these species in the physiopathology of the pulmonary infections is not known, the description of new bacterial species represents the first step to understand their possible implication in the physiopathology of the infection [4], [9].

In conclusion, the amplification of 16s DNA followed by cloning presents considerable advantages to describe and to detect new bacteria and our result clearly demonstrates that bacterial population in sputum from CF patients was complex, as the mean number of detected species increased when we sequenced more clones, and that accurate identification of these bacteria remains challenging. It is important to note that the fungi and the virus participate also effectively in these bronchial polymicrobial infections that were not studied in our work. Further studies of this complex flora by metagenomic analysis are currently needed to better understand these microbial communities, their implication in treatment and antibiotic resistance, their role in the development of chronic respiratory infections, and to identify their clinical significance in order to find new therapeutic targets.

## Materials and Methods

### Sample collection

Sputum samples from CF patients (December 2005–January 2006) were collected from two Cystic Fibrosis Treatment Centers (CFTC): children (patient <18 years), at Timone children's hospital, and adults (patient ≥18 years), at Ste. Marguerite hospital in Marseille. Sputa from patients with bronchiectasis were also collected for control analysis. All sputa were analyzed and frozen at −20°C for further study. This work has been approved by the local ethic committee of the IFR48, Faculty of Medicine, Marseille, France under the reference number 07-008. No written consent was needed for this work in accordance with the “LOI n° 2004-800 relative à la bioéthique” published in the “Journal Officiel de la République Française” the 6 August 2004 since no additional sample was taken for the study.

### Bacteriological culture and phenotypic identification

All sputa were inoculated on five agar plates including chocolate Poly ViteX agar, Columbia colistin-nalidixic acid (CNA) agar, MacConKey agar, Cepacia agar, and blood agar. All growth media were purchased from bioMérieux (bioMérieux, Marcy l'Etoile, France) and were incubated at 37°C for 48 hours except for Cepacia agar that was purchased from AES laboratory (AES, Combourg, France) and incubated at 30°C for 5 days. Colonies growing on the various media were identified using standard microbiological methods including Gram-staining, catalase and oxydase activity, susceptibility to optochin, API system (bioMérieux, Marcy l'Etoile, France), VITEK 2 Auto system (bioMérieux, Marcy l'Etoile, France), and standard procedure for antibiotic susceptibility testing. When the identification of an isolate was inaccurate (≤95% in commercial identification system) or was not in accordance with expected antibiotic susceptibility profiles, we used the amplification and sequencing of 16S rRNA gene to identify the isolates as previously described [42].

### DNA extraction

Automatic DNA extraction of sputa was performed in a MagNa Pure LC instrument (Roche Diagnostics GmbH, Mannheim, Germany) using the MagNa Pure LC DNA Isolation Kit II (Roche Diagnostics GmbH, Mannheim, Germany). One hundred microliter of each sample was treated with 200 µL of lysis buffer and 50 µL of proteinase K and was incubated overnight at 56°C as recommended by manufacturer. This mixture was shaken in a tube containing glass beads during 45 seconds then was heated at 100°C for 10 minutes. Then DNA was extracted according to the manufacturer's instruction.

### Genomic amplification

Amplification of approximately 1,000 base pairs (bp) of the 16S rRNA gene was carried out with the primer pair 536F [5′-CAGCAGCCGCGGTAATAC] and rp2 [5′-ACGGCTACCTTGTTACGACTT] (Eurogentec, Seraing, Belgium) [12]. The PCR reaction mixture (final volume, 50 µL) contained 5 µL of dNTP (2 mM of each nucleotide), 5 µL of 10× DNA polymerase buffer (QIAGEN, Courtaboeuf, France), 2 µL of MgCl_2_ (25 mM), 0.25 µL of HotStarTaq DNA polymerase (1.25 U)(QIAGEN, Courtaboeuf, France), 1 µL of each primer 536F, rp2 (10 pmol/µL), and 5 µL of extracted DNA. PCR was performed with a preliminary step at 95°C for 15 minutes followed by 35 cycles of 94°C for 1 minute, 62°C for 30 seconds, and 72°C for 1 minute and final step of extension at 72°C for 5 minutes. Then PCR products were purified using the NucleoFast® 96 PCR Kit (MACHEREY-NAGEL, Hoerdt, France) according to the manufacturer's instructions.

### Cloning procedures

Cloning of purified PCR products was performed using the pGEM® -T Easy Vector System 2 Kit (Promega, Madison, USA) as described by the manufacturer. Finally 20 µl of the bacterial suspension were resuspended in a tube containing 80 µL of LB Agar (USB, Cleveland, Ohio, USA). This suspension was plated onto LB Agar plates supplemented with Ampicilline (100 µg/mL), X-GAL (80 µg/mL), and IPTG (120 µg/mL) and the plates were incubated overnight at 37°C. Blue/white colonies were screened using these plates. Forty white colonies were collected from each plate and stored in water at −20°C.

### Insert amplification

The colonies were analysed by PCR. The reaction mixture contained 2 µL of bacterial suspension, 5 µL of dNTP (2 mM of each nucleotide), 5 µL of PCR reaction buffer (100 mM Tris-HCl, 15 mM MgCl_2_, 500 mM KCl, pH 8,3) (Roche Diagnostics GmbH, Mannheim, Germany), 0.25 µL (1.25 U) of Taq DNA polymerase (Roche Diagnostics GmbH, Mannheim, Germany) and 1 µL of each primer (10 pmol/µL) M13d [5′-GTAAAACGACGGCCAG], M13r [5′-CAGGAAACAGCTATGAC](Eurogenetec, Seraing, Belgium) in a final volume of 50 µL. The PCR mixture was preheated at 90°C for 10 minutes, followed by amplification under the following conditions: denaturation at 94°C for 50 seconds, annealing at 57°C for 1 minute, and elongation at 72°C for 2 minutes. A total of 35 cycles were performed and followed by a final elongation step at 72°C for 10 minutes. Correct sizes of the inserts were purified using the NucleoFast® 96 PCR Kit (MACHEREY-NAGEL, Hoerdt, France).

### Sequencing and informative data analysis

Purified PCR-amplified 16S rRNA inserts were sequenced in both directions using the Big Dye® Terminator V1,1 Cycle Sequencing Kit (Applied Biosystems, Courtaboeuf, France). The primers used for sequencing were M13d and M13r. The sequencing products were then run on an ABI PRISM 3130 automated sequencer (Applied Biosystems, Foster city, CA, USA). Finally the identification of bacteria was determined by comparing the obtained sequence with that of existing sequences in the GenBank database using the BLAST program available at the National Center for Biotechnology Information Web site (http://www.ncbi.nlm.nih.gov/, BLAST).

### Phylogenetic and statistical analysis

Data including the mean number of detected species per sputum (for 24 and 40 clones – in CF and control samples) and the number of different species detected in CF and bronchiectasis group were compared by chi-square test using Epi Info, version 3.4.1 (Centers for Disease Control and Prevention, Atlanta, Ga., USA). p values of ≤0.05 were considered statistically significant.
